# Trends and outcomes of trauma patients positive to marijuana and cocaine

**DOI:** 10.1007/s00068-023-02261-5

**Published:** 2023-03-31

**Authors:** José Roque-Torres, Laura Ramírez-Martínez, Ediel O. Ramos-Meléndez, Omar García-Rodríguez, Agustín Rodríguez-López, Lourdes Guerrios, Pablo Rodríguez-Ortiz

**Affiliations:** 1 School of Medicine, University of Puerto Rico Medical Sciences Campus, San Juan, Puerto Rico; 2 Trauma Surgery Division, Department of Surgery, School of Medicine, University of Puerto Rico Medical Sciences Campus, San Juan, Puerto Rico

**Keywords:** Trauma, Cocaine, Marijuana, Substance misuse

## Abstract

**Purpose:**

Substance misuse has long been recognized as a major predisposing risk factor for traumatic injury. However, there still exists no clear scientific consensus regarding the impact of drug use on patient outcomes. Therefore, this study aims to evaluate the demographic profile, hospital-course factors, and outcomes of trauma patients based on their toxicology.

**Methods:**

This is a non-concurrent cohort study of 3709 patients treated at the Puerto Rico Trauma Hospital during 2002–2018. The sample was divided into four groups according to their toxicology status. Statistical techniques used included Pearson’s chi-square test, Spearman correlation, and negative binomial and logistic regressions.

**Results:**

Admission rates for marijuana (rho = 0.87) and marijuana and cocaine positive (rho = 0.68) patients increased. Positive toxicology patients underwent surgery more often than negative testing patients (marijuana: 68.7%, cocaine: 65.6%, marijuana & cocaine: 69.8%, negative: 57.0%). Among patients with non-penetrating injuries, a positive toxicology for cocaine or marijuana was linked to a 48% and 42% increased adjusted risk of complications, 37% and 27% longer TICU LOS, and 32% and 18% longer hospital LOS, respectively.

**Conclusion:**

Our results show an association between positive toxicology for either marijuana, cocaine, or both with higher need for surgery. Additionally, our results show an increase in complications, TICU LOS, and hospital LOS among non-penetrating trauma patients testing positive for marijuana or cocaine. Therefore, this study provides valuable information on the clinical profile of patients with positive toxicology, suggesting they might benefit from more aggressive management.

## Introduction

Marijuana has recently been legalized for recreational and/or medicinal use in some states of the United States (US) and Puerto Rico (PR). Latest years have marked a significant increase in its use due to its acceptance, accessibility, and commonality among the population [1]. As of 2019, marijuana was the most widely used controlled substance in the US, with a 46.2% prevalence in ages 12 years or older [2]. Likewise, although illegal, cocaine is still one of the most used addictive drugs, with a 15.1% prevalence in ages 12 years or older [2].

Traumatic injuries remain a leading public health concern, causing over 52 million emergency visits to trauma centers in 2019 [3], and being the number one cause of death in people aged 1–44 in the US [4]. Substance misuse has long been recognized as a major predisposing risk factor of traumatic injury and recidivism [5]. Some studies report rates of positive drug screenings ranging between 30 and 40% in trauma patients [6]. Marijuana is the most prevalent drug detected in motor vehicle crash victims [7]. Meanwhile, cocaine has been linked to violent traumatic injuries [8–11].

Marijuana use may result in perturbations of cognition, perception, and psychomotor activity that, when combined with driving or other activities, is associated with mild and serious injuries [12, 13]. Cocaine also impairs the user’s judgment and motor skills and, like cannabis, heightens the susceptibility to injury and life-threatening actions [14, 15]. Both of these drugs can cause paranoia and anxiety and their use in conjunction can increase the levels of these symptoms [16]. Additionally, the use of cocaine in close temporal proximity to marijuana may prolong the effects associated with cocaine [17]. Therefore, the use of these drugs, individually or concurrently, can lead to the need for specialized treatment, emergency department visits, contraction of illnesses, and prolonged hospital stays [18].

Toxicology screening is key in understanding a patient’s medical needs and, when possible, determining whether there is an underlying substance use disorder. To date, very few studies have focused on the effects that the toxicology status has on trauma patient outcomes. Furthermore, some of the studies available show conflicting results on the subject. For example, some analyses have found an increase in mortality rates of marijuana positive patients [19], while others have reported the opposite [6] or no association [20–23]. This same pattern of results has been seen in the hospital length of stay (LOS) [20, 22–28], the Injury Severity Score (ISS) [19, 26], and other important parameters. Due to this lack of studies and the conflicting results from the available ones, there exists no clear scientific consensus regarding the impact of drug use on trauma patient outcomes. This fact, along with the increase in marijuana and cocaine availability and consumption, emphasizes the need for further research focusing on this topic.

Therefore, our study seeks to fill this gap in knowledge by examining the trends in trauma admission rates of toxicology-positive patients during 2002–2018; as well as analyzing the association between toxicology testing status and the demographic profile, injury-related and hospital course factors, and outcomes in trauma patients.

## Methods

### Study design and setting

This is a non-concurrent cohort study of patients admitted to the Puerto Rico Trauma Hospital (PRTH), a level II trauma center that serves as the major referral hospital for polytrauma patients in PR and the Caribbean. Our 92-bed hospital owns a trauma registry that is part of the US National Trauma Registry System, which was used as the data source for the present study. This registry is managed by specialized and trained personnel and is subject to a quarterly quality–control review according to the standards and requirements of the American College of Surgeons.

### Study population

**Fig. 1:**
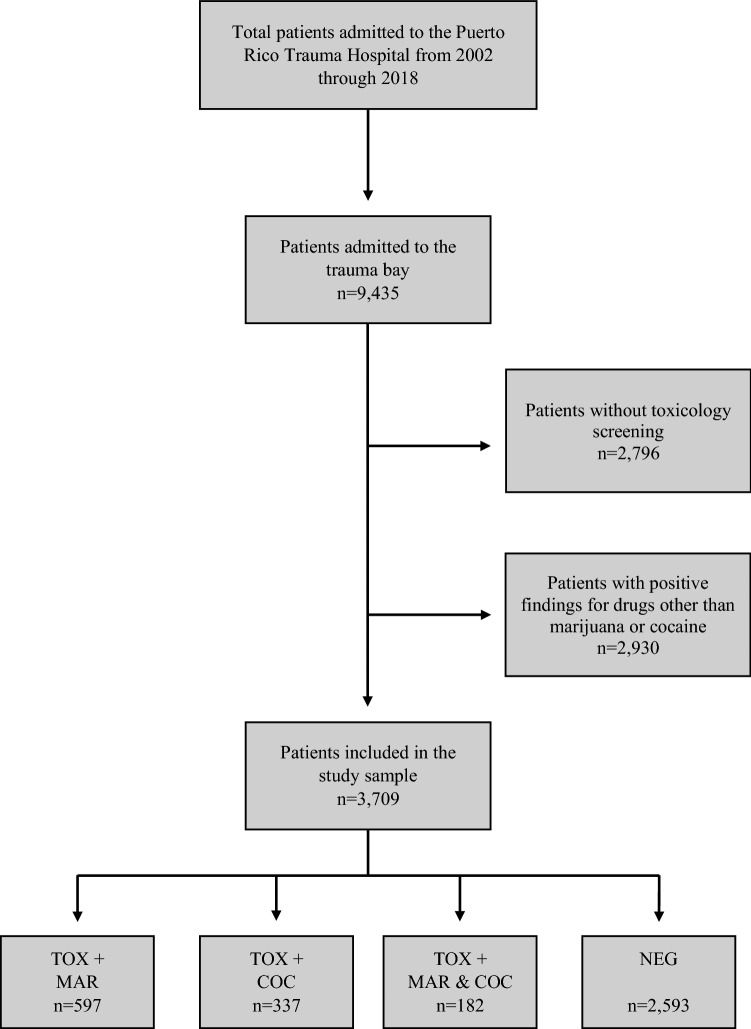
Flowchart of the study sample selection. Note. *TOX + * positive test, *NEG* negative test, *MAR* marijuana, *COC* cocaine

A total of 3709 patients treated at the PRTH from 2002 through 2018 and who met inclusion criteria were chosen for this study. The eligibility criteria consisted of admissions to the trauma bay with a known toxicology testing status, whether negative or positive for marijuana, cocaine, or both. Those patients who did not have a toxicology screening, as well as those with positive findings for drugs other than marijuana or cocaine were excluded from the dataset. Furthermore, individuals who tested positive for marijuana or cocaine in combination with any other drug were also excluded. We excluded this particular subgroup of patients in order to minimize the effects of confounding –since the influence of marijuana and/or cocaine on trauma outcomes, when combined with other drugs might be different from that of marijuana and/ or cocaine alone-. This restriction directly translate into an increased internal validity of our study. The effective sample was divided into 4 groups according to toxicology testing status: positive for marijuana (MAR), positive for cocaine (COC), positive for both marijuana and cocaine (MAR & COC), and negative (NEG). Figure 1 depicts a flowchart of the selection of the study sample.

### Variables

Demographic, injury-related, hospital-based, and outcome variables were measured. Patient characteristics included sex, age, and health insurance status. Injury-related factors of interest comprised type of injury, mechanism of injury, vital signs recorded upon admission (i.e., respiratory rate, systolic blood pressure, temperature, and heart rate), ISS, and Glasgow Coma Scale (GCS). Regarding the hospital course and outcomes, we collected data on the need for surgery, mechanical ventilation (MV), days on MV, trauma intensive care unit (TICU) admission, and TICU LOS, hospital LOS, complications, and in-hospital mortality. Complications considered were as follows: airway, pulmonary, cardiac, gastrointestinal, hepatic/pancreatic/biliary/splenic, hematologic, infectious, renal/genitourinary, musculoskeletal/skin, neurologic, and vascular.

### Statistical analysis

All descriptive values are presented as median with interquartile range (IQR), or absolute (*n*) and relative (*%*) frequencies, as appropriate. Comparisons of categorical data were conducted using the Pearson’s chi-square test or Fisher exact test, as applicable; while contrasts of continuous data were done via the Kruskal–Wallis test and post hoc Dunn’s test with Bonferroni adjustment for multiple comparisons (i.e., the observed p value was multiplied by the number of comparisons made). On the other hand, Spearman's rank correlation coefficient was used to detect monotonic trends in admission rates of toxicology-positive patients over time.

As for multivariate testing, zero-truncated negative binomial regressions were used to model TICU LOS and hospital LOS as a function of toxicology testing status and covariates. Furthermore, the effect of the principal explanatory variable on the development of complications and in-hospital death was modeled with unconditional logistic regressions. All models are adjusted for those demographic and injury-related factors that were statistically significant in bivariate analysis. And the examination of first-order interaction terms between the exposure (i.e., toxicology testing status) and modifying variables were explored using the likelihood ratio test.

For all analyses, the level of significance was set at a two-sided p value < 0.05. The statistical software used to conduct the analyses was STATA version 14 (STATA Corp, College Station, TX, USA). This study was approved by the Institutional Review Board of the Medical Sciences Campus of the University of Puerto Rico and a waiver of consent was obtained.

## Results

Of the total number of patients admitted to the Stabilization Unit of the PRTH during the seventeen-year study period and met inclusion criteria, 1116 (30.1%) patients were positive for marijuana, cocaine, or both upon admission. Marijuana was detected in 597 (16.1%), followed by cocaine in 337 (9.1%), and marijuana and cocaine combined in 182 (4.9%) (See Fig. 1).

**Fig. 2:**
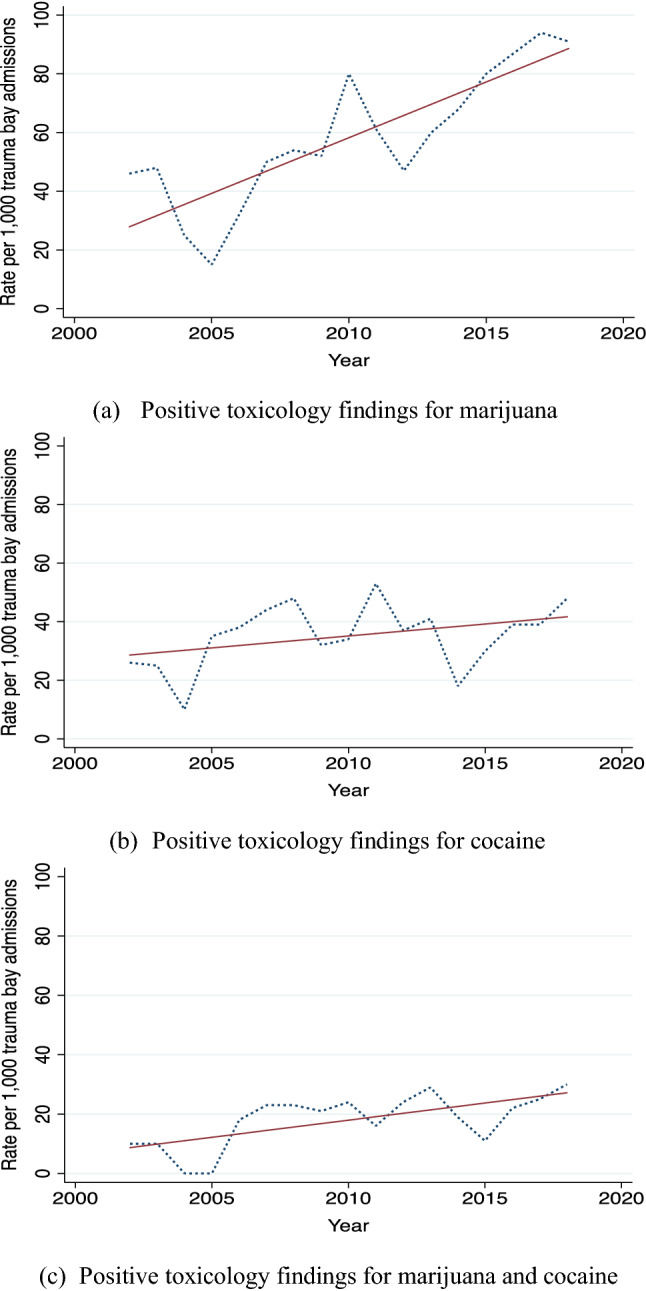
(Three panels) Trends in injured patients with positive toxicology findings admitted to the Puerto Rico Trauma Hospital from 2002 to 2018

Admission rates of marijuana positive patients to the trauma bay significantly increased from 46 to 91 cases per 1000 admissions between 2002 and 2018 (rho = 0.87; *p* < *0.001*). Similarly, admission rates of those patients with a positive toxicology for marijuana and cocaine notably rose from 10 to 30 cases per 1000 admissions to the trauma bay within the same time frame (rho = 0.68; *p* = *0.003*). However, there was no statistically significant trend in admissions of cocaine positive patients over the time-period studied (rho = 0.40; *p* = *0.113*) (See Fig. 2).

As for the sociodemographic profile, positive toxicology groups showed a higher prevalence of males, with the marijuana and cocaine positive group having the highest percentage of them (MAR: 90.1%, COC: 91.7%, MAR & COC: 96.2%, NEG: 77.7%; *p* < *0.001*). Additionally, patients who tested positive for marijuana were predominantly between 18 and 24 years old (36.4%), while those who tested positive for marijuana and cocaine were mainly between 25 and 34 years (36.3%), and cocaine positive patients as well as negative patients had the highest relative frequency among 35–44 years (27.2%) and > 54 years (27.2%), respectively (*p* < *0.001*). Furthermore, the proportion of patients who reported lack of health insurance coverage was higher among the positive toxicology patients than among the negative ones (MAR: 12.7%, COC: 14.4%, MAR & COC: 18.9%, NEG: 8.3%; *p* < *0.001*).

Patients testing positive in the screening test exhibited a higher prevalence of penetrating trauma as compared with patients who tested negative, with the marijuana and the marijuana and cocaine positive groups hovering around 50% (MAR: 49.4%, COC: 38.2%, MAR & COC: 54.4%, NEG: 21.5%; *p* < *0.001*). In terms of the mechanisms of injury, gunshot wounds were more common among those with a positive toxicology (MAR: 44.9%, COC: 27.9%, MAR & COC: 44.5%, NEG: 17.9%), whereas motor vehicle accidents occurred more frequently among their negative toxicology counterparts (MAR: 32.0%, COC: 25.8%, MAR & COC: 23.1%, NEG: 41.5%). Stab wounds were also seen more often in the cocaine and the marijuana and cocaine positive groups in comparison with the others (MAR: 5.2%, COC: 10.1%, MAR & COC: 9.3%, NEG: 4.1%). Interestingly, the proportion of pedestrians in the cocaine positive (15.7%) and the negative (14.9%) groups was roughly double that of the marijuana (7.5%) and the marijuana and cocaine positive (8.8%) groups (*p* < *0.001*).

Concerning the injury severity of patients, the ISS value was distributed differently between groups, as patients with positive toxicology findings had higher relative frequencies of an ISS ≥ 25 (MAR: 27.4%, COC: 25.7%, MAR & COC: 28.0%, NEG: 23.4%; *p* = *0.001*); while the GCS was not statistically linked to toxicology status (*p* > *0.05*).

Regarding hospital course measures, patients in all three positive toxicology groups required surgery more often than patients with negative results (MAR: 68.7%, COC: 65.6%, MAR & COC: 69.8%, NEG: 57.0%; *p* < *0.001*). Additionally, the median [IQR] TICU LOS of patients with cocaine positive toxicology (17.5 [20.5] days) was significantly longer than that of patients with negative toxicology (13 [19] days; Bonferroni post hoc *p* = *0.013*) and that of subjects with marijuana positive results (11 [20] days; Bonferroni post hoc *p* = *0.007*). This group also spent more days on MV (13 [21] days) than the marijuana positive (9 [17] days; Bonferroni post hoc *p* = *0.006*) and the negative toxicology groups (11 [20] days; Bonferroni post hoc *p* = *0.060*). Admission to TICU, need for MV, and hospital LOS were not associated with toxicology results (*p* > *0.05*) (See Table 1).

**Table 1:** Sociodemographic characteristics, injury profile, hospital course, and outcomes of patients admitted to the Puerto Rico Trauma Hospital according to the toxicology testing status (N = 3709)

Characteristic	Total cohort (N = 3709) n (%)	MAR (n = 597) n (%)	COC (n = 337) n (%)	MAR & COC (n = 182) n (%)	NEG (n = 2593) n (%)	*p* value
Sociodemographic data						
Sex	(n = 3707)	(n = 597)	(n = 337)	(n = 182)	(n = 2591)	< 0.001
Male	3036 (81.9)	538 (90.1)	309 (91.7)	175 (96.2)	2014 (77.7)	
Female	671 (18.1)	59 (9.9)	28 (8.3)	7 (3.8)	577 (22.3)	
Age, years	(n = 3702)	(n = 597)	(n = 335)	(n = 182)	(n = 2588)	< 0.001
< 18	490 (13.2)	87 (14.6)	3 (0.9)	1 (0.6)	399 (15.4)	
18–24	658 (17.8)	217 (36.4)	34 (10.2)	43 (23.6)	364 (14.1)	
25–34	856 (23.1)	184 (30.8)	85 (25.4)	66 (36.3)	521 (20.1)	
35–44	491 (13.3)	49 (8.2)	91 (27.2)	43 (23.6)	308 (11.9)	
45–54	418 (11.3)	27 (4.5)	72 (21.5)	26 (14.3)	293 (11.3)	
> 54	789 (21.3)	33 (5.5)	50 (14.9)	3 (1.7)	703 (27.2)	
Insurance status	(n = 3680)	(n = 589)	(n = 333)	(n = 180)	(n = 2578)	< 0.001
Private insurance	2168 (58.9)	228 (38.7)	158 (47.5)	59 (32.8)	1723 (66.8)	
Public insurance	1141 (31.0)	286 (48.6)	127 (38.1)	87 (48.3)	641 (24.9)	
Uninsured	371 (10.1)	75 (12.7)	48 (14.4)	34 (18.9)	214 (8.3)	
Injury-related data						
Type of Injury	(n = 3703)	(n = 597)	(n = 335)	(n = 182)	(n = 2589)	< 0.001
Non-Penetrating	2623 (70.8)	302 (50.6)	207 (61.8)	83 (45.6)	2031 (78.5)	
Penetrating	1080 (29.2)	295 (49.4)	128 (38.2)	99 (54.4)	558 (21.5)	
Mechanism of injury	(n = 3709)	(n = 597)	(n = 337)	(n = 182)	(n = 2593)	< 0.001
MVA	1395 (37.6)	191 (32.0)	87 (25.8)	42 (23.1)	1075 (41.5)	
GSW	907 (24.5)	268 (44.9)	94 (27.9)	81 (44.5)	464 (17.9)	
SW	189 (5.1)	31 (5.2)	34 (10.1)	17 (9.3)	107 (4.1)	
Falls	441 (11.9)	34 (5.7)	33 (9.8)	13 (7.1)	361 (13.9)	
Pedestrians	499 (13.5)	45 (7.5)	53 (15.7)	16 (8.8)	385 (14.9)	
Others	278 (7.5)	28 (4.7)	36 (10.7)	13 (7.1)	201 (7.8)	
Respiratory rate	(n = 3642)	(n = 578)	(n = 328)	(n = 179)	(n = 2557)	0.164
< 12 rpm	79 (2.2)	16 (2.8)	10 (3.1)	4 (2.2)	49 (1.9)	
12–20 rpm	1611 (44.2)	229 (39.6)	150 (45.7)	75 (41.9)	1157 (45.3)	
> 20 rpm	1952 (53.6)	333 (57.6)	168 (51.2)	100 (55.9)	1351 (52.8)	
Systolic blood pressure	(n = 3689)	(n = 592)	(n = 335)	(n = 182)	(n = 2580)	0.013
< 90 mmHg	326 (8.8)	43 (7.3)	45 (13.4)	15 (8.2)	223 (8.6)	
≥ 90 mmHg	3363 (91.2)	549 (92.7)	290 (86.6)	167 (91.8)	2357 (91.4)	
Temperature	(n = 3634)	(n = 576)	(n = 331)	(n = 181)	(n = 2546)	0.092^a^
< 95.1 F	412 (11.3)	61 (10.6)	50 (15.1)	21 (11.6)	280 (11.0)	
95.1 F–100.8 F	3191 (87.8)	509 (88.4)	275 (83.1)	160 (88.4)	2247 (88.3)	
> 100.8 F	31 (0.9)	6 (1.0)	6 (1.8)	0 (0)	19 (0.8)	
Heart rate	(n = 3701)	(n = 597)	(n = 336)	(n = 182)	(n = 2586)	0.005
< 60 bpm	135 (3.7)	33 (5.5)	16 (4.8)	9 (5.0)	77 (3.0)	
60–100 bpm	1914 (51.7)	311 (52.1)	179 (53.3)	107 (58.8)	1317 (50.9)	
> 100 bpm	1652 (44.6)	253 (42.4)	141 (42.0)	66 (36.3)	1192 (46.1)	
Injury severity score	(n = 3690)	(n = 596)	(n = 335)	(n = 182)	(n = 2577)	0.001
1–9	1036 (28.1)	148 (24.8)	89 (26.6)	56 (30.8)	743 (28.8)	
10–15	706 (19.1)	146 (24.5)	63 (18.8)	33 (18.1)	464 (18.0)	
16–24	1046 (28.4)	139 (23.3)	97 (29.0)	42 (23.1)	768 (29.8)	
≥ 25	902 (24.4)	163 (27.4)	86 (25.7)	51 (28.0)	602 (23.4)	
Glasgow coma scale	(n = 3687)	(n = 595)	(n = 334)	(n = 179)	(n = 2579)	0.173
13–15	2813 (76.3)	454 (76.3)	242 (72.5)	145 (81.0)	1972 (76.5)	
9–12	253 (6.9)	33 (5.6)	32 (9.6)	11 (6.2)	177 (6.9)	
≤ 8	621 (16.8)	108 (18.2)	60 (18.0)	23 (12.9)	430 (16.7)	
Hospital course and outcome data						
Surgery required						< 0.001
Yes	2235 (60.3)	410 (68.7)	221 (65.6)	127 (69.8)	1477 (57.0)	
Admission to TICU						0.244
Yes	1146 (30.9)	171 (28.6)	116 (34.4)	51 (28.0)	808 (31.2)	
TICU LOS, days						0.015
Median (IQR)	13 (19)	11 (20)	17.5 (20.5)	14 (16)	13 (19)	
MV Required						0.095
Yes	1320 (35.6)	199 (33.3)	139 (41.3)	61 (33.5)	921 (35.5)	
MV, days						0.019
Median (IQR)	11 (18)	9 (17)	13 (21)	12 (17)	11 (20)	
Hospital LOS, days						0.181
Median (IQR)	11 (20)	10 (17)	12 (27)	9 (16)	11 (21)	
In-hospital Mortality						0.001
Alive	3148 (84.9)	533 (89.3)	278 (82.5)	163 (89.6)	2174 (83.8)	
Dead	561 (15.1)	64 (10.7)	59 (17.5)	19 (10.4)	419 (16.2)	

*MAR* marijuana, *COC* cocaine, *NEG* negative test, *IQR* interquartile range, *MVA* motor vehicle accident, *GSW* gunshot wound, *SW* stab wound, *TICU* trauma intensive care unit, *LOS* length of stay, *MV* mechanical ventilation

^a^Fisher’s exact test

The distribution of in-hospital complications among the study groups showed that respiratory failure (MAR: 3.9%, COC: 11.6%, MAR & COC: 7.1%, NEG: 9.5%; p < 0.001) and shock (MAR: 1.0%, COC: 3.9%, MAR & COC: 1.1%, NEG: 0.9%; p < 0.001) were more frequent among those with a cocaine positive toxicology result. The highest relative frequency of renal failure was also seen among patients who were positive for cocaine, whereas those who were positive for marijuana showed the lowest one (MAR: 0.7%, COC: 3.6%, MAR & COC: 2.8%, NEG: 2.4%; p = 0.020). Nevertheless, this latter group exhibited the highest percent of intra-abdominal abscesses (MAR: 2.9%, COC: 0.6%, MAR & COC: 0.6%, NEG: 1.5%; p = 0.023) (See Table 2).

**Table 2:** In-hospital complications of patients admitted to the Puerto Rico Trauma Hospital according to the toxicology testing status (N = 3709)

Complication	Total Cohort (N = 3709) n (%)	MAR (n = 597) n (%)	COC (n = 337) n (%)	MAR & COC (n = 182) n (%)	NEG (n = 2,593) n (%)	p value
Pulmonary						
ARDS	150 (4.0)	26 (4.4)	22 (6.5)	8 (4.4)	94 (3.6)	0.081
Pneumonia	344 (9.3)	48 (8.0)	39 (11.6)	17 (9.3)	240 (9.3)	0.362
Atelectasis	39 (1.1)	9 (1.5)	3 (0.9)	0 (0)	27 (1.0)	0.399^a^
Hemothorax	63 (1.7)	12 (2.0)	11 (3.3)	3 (1.7)	37 (1.4)	0.092
Respiratory Failure	320 (8.6)	23 (3.9)	39 (11.6)	13 (7.1)	245 (9.5)	< 0.001
Pleural Effusion	87 (2.4)	10 (1.7)	10 (3.0)	5 (2.8)	62 (2.4)	0.595
Cardiovascular						
Arrhythmia	69 (1.9)	11 (1.8)	10 (3.0)	2 (1.1)	46 (1.8)	0.400
Cardiac Arrest	109 (2.9)	15 (2.5)	9 (2.7)	5 (2.8)	80 (3.1)	0.877
Shock	43 (1.2)	6 (1.0)	13 (3.9)	2 (1.1)	22 (0.9)	< 0.001^a^
Infectious						
Cellulitis/Traumatic Injury	50 (1.4)	5 (0.8)	8 (2.4)	1 (0.6)	36 (1.4)	0.229^a^
Intra-Abdominal Abscess	59 (1.6)	17 (2.9)	2 (0.6)	1 (0.6)	39 (1.5)	0.023
Sepsis-Like Syndrome	61 (1.6)	9 (1.5)	8 (2.4)	4 (2.2)	40 (1.5)	0.640
Septicemia	36 (1.0)	4 (0.7)	6 (1.8)	1 (0.6)	25 (1.0)	0.394^a^
Wound Infection	49 (1.3)	10 (1.7)	5 (1.5)	1 (0.6)	33 (1.3)	0.708^a^
Sepsis	52 (1.4)	6 (1.0)	7 (2.1)	0 (0)	39 (1.5)	0.198^a^
CRBI	70 (1.9)	7 (1.2)	5 (1.5)	1 (0.6)	57 (2.2)	0.168
Renal/Genitourinary						
Renal Failure	82 (2.2)	4 (0.7)	12 (3.6)	5 (2.8)	61 (2.4)	0.020
Urinary Tract Infection	128 (3.5)	14 (2.4)	13 (3.9)	8 (4.4)	93 (3.6)	0.393
Musculoskeletal/Skin						
ECS	53 (1.4)	8 (1.3)	7 (2.1)	1 (0.6)	37 (1.4)	0.617
Pressure Ulcer	97 (2.6)	13 (2.2)	16 (4.8)	3 (1.7)	65 (2.5)	0.064

In-hospital complications displayed in the table were selected based on their unconditional relative frequency (≥ 1%)

*MAR* marijuana, *COC* cocaine, *NEG* negative test, *ARDS* Acute Respiratory Distress Syndrome, *CRBI* Catheter Related Blood Infection, *ECS* Extremity Compartment Syndrome

^a^Fisher’s exact test

When evaluating the magnitude of the association of the toxicology testing status with the study outcomes, a positive cocaine result was linked to a 35% (OR = 1.35; 95% CI: 1.07–1.71) higher unadjusted odds of developing complications, a 34% (RR = 1.34; 95% CI: 1.10–1.63) longer TICU LOS, and a 30% (RR = 1.30; 95% CI: 1.12–1.51) longer hospital LOS. However, in the multivariate analysis, the type of trauma was found to modify the effect of the toxicology testing status on these three endpoints and, therefore, findings are displayed separately for non-penetrating trauma patients and for penetrating trauma patients.

Among non-penetrating trauma patients, the adjusted odds of developing in-hospital complications were 48% (AOR = 1.48; 95% CI: 1.07–2.04) higher for those who were positive for cocaine and 42% (AOR = 1.42; 95% CI: 1.07–1.88) higher for those who were positive for marijuana when compared to those who tested negative. Similarly, the TICU LOS was 37% (ARR = 1.37; 95% CI: 1.10–1.71) longer for non-penetrating trauma patients who tested positive for cocaine and 27% (ARR = 1.27; 95% CI: 1.04–1.55) longer for those who tested positive for marijuana than for their counterparts with negative results. And the hospital LOS, among subjects with non-penetrating injuries, was 32% (ARR = 1.32; 95% CI: 1.11–1.57) longer for those who tested positive for cocaine and 18% (ARR = 1.18; 95% CI: 1.02–1.37) longer for those who tested positive for marijuana when compared to those who tested negative. Nevertheless, no significant associations were found between the toxicology testing status and these three outcomes in penetrating trauma patients, according to the multivariate modeling (See Table 3).

**Table 3:** Estimates of the effect of the toxicology testing status on development of complications, TICU LOS, and hospital LOS, stratified by type of trauma

Outcome	Overall trauma	Penetrating trauma		Non-penetrating trauma	
	OR^*^/RR^ꜧ^ (95% CI)	OR^*^/RR^ꜧ^ (95% CI)	AOR^*^/ARR^ꜧ^ (95% CI)	OR^*^/RR^ꜧ^ (95% CI)	AOR^*^/ARR^ꜧ^ (95% CI)
≥ 1 complications					
MAR	0.86 (0.70–1.05)^*^	0.67 (0.49–0.93)^*^	0.70 (0.49–1.00)^*a^	1.05 (0.81–1.37)^*^	1.42 (1.07–1.88)^*a^
COC	1.35 (1.07–1.71)^*^	1.04 (0.69–1.57)^*^	0.91 (0.58–1.43)^*a^	1.58 (1.18–2.12)^*^	1.48 (1.07–2.04)^*a^
MAR & COC	1.02 (0.74–1.41)^*^	0.96 (0.60–1.53)^*^	1.03 (0.63–1.67)^*a^	1.07 (0.67–1.72)^*^	1.17 (0.70–1.94)^*a^
NEG	Reference	Reference	Reference	Reference	Reference
TICU LOS					
MAR	0.93 (0.79–1.10)^ꜧ^	0.82 (0.61–1.10)^ꜧ^	0.91 (0.67–1.24)^ꜧb^	1.01 (0.82–1.26)^ꜧ^	1.27 (1.04–1.55)^ꜧb^
COC	1.34 (1.10–1.63)^ꜧ^	1.10 (0.76–1.57)^ꜧ^	0.97 (0.67–1.40)^ꜧb^	1.46 (1.15–1.86)^ꜧ^	1.37 (1.10–1.71)^ꜧb^
MAR & COC	1.12 (0.84–1.49)^ꜧ^	1.06 (0.67–1.66)^ꜧ^	1.15 (0.74–1.80)^ꜧb^	1.17 (0.79–1.72)^ꜧ^	1.25 (0.87–1.81)^ꜧb^
NEG	Reference	Reference	Reference	Reference	Reference
Hospital LOS					
MAR	0.95 (0.84–1.07)^ꜧ^	0.83 (0.68–1.01)^ꜧ^	0.87 (0.71–1.07)^ꜧc^	1.07 (0.92–1.25)^ꜧ^	1.18 (1.02–1.37)^ꜧc^
COC	1.30 (1.12–1.51)^ꜧ^	0.96 (0.73–1.26)^ꜧ^	0.90 (0.69–1.18)^ꜧc^	1.52 (1.27–1.83)^ꜧ^	1.32 (1.11–1.57)^ꜧc^
MAR & COC	0.94 (0.77–1.15)^ꜧ^	0.94 (0.69–1.27)^ꜧ^	0.94 (0.70–1.26)^ꜧc^	0.96 (0.72–1.27)^ꜧ^	0.91 (0.70–1.19)^ꜧc^
NEG	Reference	Reference	Reference	Reference	Reference

*MAR* marijuana, *COC* cocaine, *NEG* negative test, *TICU* trauma intensive care unit, *LOS* length of stay, *OR* odds ratio, *RR* rate ratio, *AOR* adjusted odds ratio, *ARR* adjusted rate ratio, *CI* confidence interval

* OR/AOR, ꜧ RR/ARR

^a^Adjusted for sex, age, health insurance, heart rate, temperature, Injury Severity Score, and Glasgow Coma Scale

^b^Adjusted for age, systolic blood pressure, Injury Severity Score, and Glasgow Coma Scale

^c^Adjusted for age, health insurance, heart rate, Injury Severity Score, and Glasgow Coma Scale

**Fig. 3:**
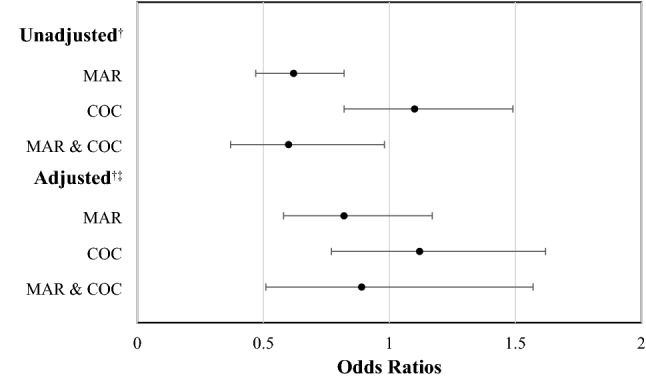
Univariate and multivariate analyses for the risk of death according to toxicology testing status. *MAR* marijuana, *COC* cocaine; †: patients with a negative toxicology test result were used as the reference group; ‡: values were obtained after adjusting for the following confounders: age, health insurance, mechanism of injury, temperature, systolic blood pressure, Injury Severity Score, and Glasgow Coma Scale

In-hospital mortality, despite being strongly related to toxicology status in the bivariate analysis (*p* = *0.001*), with patients testing positive for marijuana (10.7%) or for marijuana and cocaine (10.4%) having the lowest death rates and those testing positive for cocaine (17.5%) or testing negative (16.2%) experiencing the highest ones (See Table 1); lost its statistical significance after adjusting for confounders (See Fig. 3).

## Discussion

Misuse and abuse of illicit drugs have increased worldwide, and consequently so has the risk of trauma-related injuries [11, 29–31]. Multiple studies have focused on the effects of marijuana and cocaine on trauma patients [5, 6, 11, 20, 24, 30]. However, when analyzing the effects on patient outcomes, they have failed to reach a consensus. This study evaluated the association between the toxicology testing status of patients admitted to the PRTH and the demographic profile, injury-related and hospital course factors, and outcomes of the trauma patients.

Our analysis of the trends of admission rates for toxicology-positive patients during 2002–2018 demonstrated a significant increase in the number of patients testing positive for marijuana, and marijuana and cocaine. Meanwhile, the number of patients testing positive for cocaine did not show any statistically significant change. These results are consistent with the recent increase in marijuana use in the US population reported by the National Institute on Drug Abuse (NIDA) along with previous studies [25, 32, 33]. However, NIDA also reported a diminution of cocaine use since 2007, which is not seen in our admissions trends [32].

In the US, marijuana use is most prevalent among the 18–25 age range, while cocaine is most prevalent among the 26 or older age range [4]. Our results show a similar distribution with patients testing positive for marijuana predominantly being in the 18–24 age group, and patients testing positive for cocaine primarily being in the 35–44 group. These results were compared to the drug use statistics of the US mainland since there was a lack of statistics on adult marijuana and cocaine use in PR. Additionally, our analysis shows that the patients who tested positive for both marijuana and cocaine were mainly in the 25–34 age group. This age group is located between the most prevalent age groups of marijuana positive and cocaine positive patients.

Our analysis suggested that the use of marijuana and cocaine, independently and in conjunction, is linked to interpersonal violence (i.e., gunshot wounds, stab wounds). On the other hand, a negative toxicology test was associated with non-violent mechanisms of injury (i.e., motor vehicle accidents). These findings are in agreement with previous studies, which links marijuana and cocaine to violent injury mechanisms of trauma [8, 9, 30, 34]. Additionally, recent literature on the topic has found an increase in marijuana positive toxicology results among patients in motor vehicle accidents [12, 13]. Our results seem to confirm this trend since motor vehicle accidents were the second most common mechanism of injury for patients who tested positive to marijuana.

In terms of the patients’ clinical profile, the ISS varied depending on the toxicology test result. Patients with a positive toxicology test had higher relative frequencies of an ISS ≥ 25. These results are in agreement with a previous study from Spain, which reported that the pre-injury consumption of drugs increases the likelihood of more severe injuries [19]. However, another study did show that cocaine positive patients had a lower ISS than negative patients [26]. Perhaps these observed results are related to the difference in the mechanisms of injury of patients with a positive toxicology and those with a negative toxicology. As previously mentioned, our study found a link between a positive toxicology and violence related mechanisms of injury, like gunshot wounds and stab wounds, which constitute penetrating injuries. Meanwhile, our data showed that a negative toxicology was associated with non-violent mechanisms of injury, like motor vehicle accidents, which tend to cause non-penetrating injuries.

Our study showed that surgery was required significantly more often for patients in all three positive groups when compared to patients in the negative group. A previous study on marijuana positive patients found an increase in the number of surgeries required, when compared to negative patients [27]. Meanwhile, a study on cocaine positive patients found no change in the need for surgery, when compared to patients testing negative [28]. Unfortunately, we were not able to find previous studies that focused on patients testing positive for both marijuana and cocaine simultaneously for comparison.

Furthermore, our analysis revealed that admissions to the TICU were not associated with toxicology results. Previous studies on the matter showed conflicting evidence. One reported similar results to ours in patients testing positive to cocaine [20], while another one pointed out to an increase in TICU admissions for cocaine or marijuana positive patients [28]. We also found that the need for MV was not linked to toxicology results. Preceding research had found that patients with a positive toxicology result required MV more often than patients testing negative [6, 21, 35]. Regarding the total amount of days receiving MV, previous research showed no differences between marijuana positive and negative testing patients [24]. These results were also seen in our data, nevertheless we also found that the cocaine positive group spent more days on MV than patients in the marijuana positive and negative groups.

When analyzing the study endpoints, the type of trauma modified the effect of toxicology status on complications, TICU LOS, and hospital LOS. We found that, among non-penetrating trauma patients, a positive toxicology for marijuana or cocaine was related to an increased adjusted risk of complications, longer TICU LOS, and longer hospital LOS; while in penetrating trauma patients no significant associations were found. To our knowledge these particular findings have not been reported in previous studies, which emphasizes the need for further research into how the mechanisms of injury affect the outcomes of patients who test positive for these drugs.

The published literature has examined these outcomes without considering interactions with the type of trauma, and most of the studies have used bivariate statistical techniques (an approach that does not account for confounding factors). One of the widest studied parameters is the hospital LOS, which has yielded inconsistent results. Some works showed no difference in hospital LOS for marijuana or cocaine positive patients when compared to negative testing patients [22–24, 26, 28]. Meanwhile, other analyses did find that patients with positive toxicology had longer hospital LOS than negative patients [20, 25, 27].

There is still conflicting evidence on the effects of drugs on mortality rates of trauma patients. For example, in marijuana positive patients, one study showed lower mortality rates [6], another reported an increase in mortality rates [19], and others found no association between marijuana and the risk of in-hospital mortality rates in trauma patients [20–23]. In cocaine patients, the majority of studies have found no association between having a cocaine positive result and the risk of in-hospital mortality [20, 22, 26, 28]. However, there was one study that did find an increase in mortality rates for patients with a positive toxicology test [19]. Nevertheless, after adjusting for confounders, our results showed no significant difference in the in-hospital mortality rate for the different groups, meaning that we found no association between a positive toxicology result and a change in the mortality rates of our patients.

The use of marijuana and cocaine both independently and in conjunction has direct effects on a person’s physiology, psychology, and behavior [8, 9]. While the psychological effects of marijuana vary from person to person, these effects may include paranoia, delusions, and hallucinations [36–38]. Meanwhile, cocaine’s effects can also include panic and paranoia, as well as euphoria and irritability [39–41]. These effects can all lead to erratic and violent behaviors, which can all translate to traumatic injuries. Therefore, as illicit drug use continues to increase, it is extremely important to understand the trends shown by these patients. This explains the relevance of our results, along with the need for further studies on this topic, in order to reach a consensus on the effects these drugs have on trauma patients. This will lead to a better understanding of their condition, which will allow for the development of intervention strategies that improve their outcomes. Additionally, trauma is a preventable disease, which means that strategies can also be established to promote the safety of marijuana and cocaine users.

Due to its retrospective nature, our study has some limitations. First, it did not allow us to analyze some confounding variables (e.g., time of drug consumption, amount of drug consumed, among others) that could have impacted the effects the drugs had on the patients. Additionally, due to the half-life of the metabolites used to measure marijuana and cocaine use, there is sometimes a challenge in detecting the presence of these drugs. Therefore, there is a possibility that some patients with a negative toxicology result could have had these drugs present in their bodies during the injury. Furthermore, our study did not focus on the specific physiological effects of the consumption of marijuana and cocaine that could have impacted the outcomes of patients. Also, our study did not focus on the difference between sporadic recreational and constant medical use of marijuana in these patients, which could also be important in explaining our results. Besides, most of the data collected for the study was obtained before the legalization of medical marijuana use in PR. Hence, we were not able to examine for any post-legalization exclusive changes in the trends and outcomes of these patients. Lastly, the data collected was limited to a local population in a single institution, which means that our results cannot necessarily be extrapolated to other populations. However, the PRTH is the only trauma specialized hospital on the island. In addition, since alcohol tests were not included in our data set, we were not able to address this variable in our study. Despite these limitations, this study is one of the first to evaluate drug use in trauma patients in PR, and one of the first to include patients who use marijuana and cocaine simultaneously, providing the precedent for future studies, which might also explore interactions between marijuana and cocaine and other drugs.

In summary, our study revealed an association between a positive toxicology test in trauma patients and a higher need for surgery. Additionally, we found that a positive toxicology for marijuana or cocaine was linked to an increased adjusted risk of complications, longer TICU LOS, and longer hospital LOS in non-penetrating trauma patients. These findings suggest that patients with positive toxicology results, particularly those with non-penetrating trauma, might benefit from more aggressive management. However, there still exist conflicting results regarding this topic, and for this reason, we recommend more in-depth research on how the physiology of trauma patients is affected by these drugs. This will help reach a consensus on the topic, which is essential to identify the most effective treatments for patients that have a positive toxicology test.

## Data Availability

Data and code availability not accessible.
